# Letter to the editor: ocular surface injury following alcohol-based hand sanitizer use in Covid-19 prevention

**DOI:** 10.5935/0004-2749.202100105

**Published:** 2021

**Authors:** Cristiano Urbano Becker, Gustavo Salomão, Myrna Serapião, Alexandre Manetta, Heloísa Nascimento, Rubens Belfort Junior

**Affiliations:** 1 Instituto da Visão, São Paulo, SP, Brazil; 2 Faculdade de Medicina do ABC, São Paulo, SP, Brazil; 3 Escola Paulista de Medicina, Universidade Federal de São Paulo, São Paulo, SP, Brazil

Dear Editor,

The identification of SARS-CoV-2 has led to severe worldwide public health challenges^(1)^. The pandemic has greatly increased the demand for hand disinfectants, especially for alcohol-based hand sanitizers (ABHS)^(2)^.

Recent studies have revealed that ABHS inactivate SARS-CoV-2; thus, their use has been recommended by the Centers for Disease Control (CDC)^(3)^. However, the development of different types of hand sanitizer and the growing demand for these products have raised safety concerns. To facilitate their use, ABHS are often provided in dispensers with a pedal at a convenient height that can be reached by most adults, usually at 100 cm above the floor. We report two children who presented with ocular surface injury following the inappropriate use of these ABHS hand dispensers.

**Figure 1:**
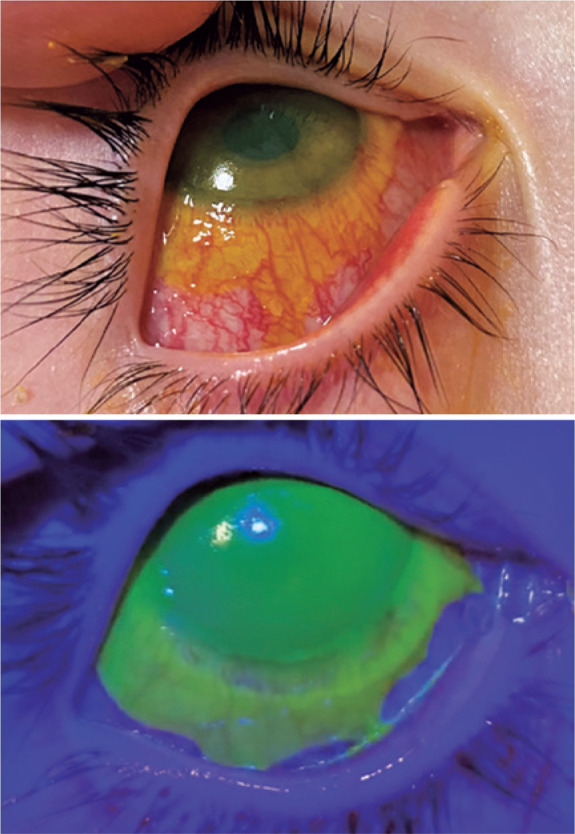
Patient 1 Right eye in the second day, showing corneoconjunctival large ulcer after topical fluoresceine 2% instilation.

**Figure 2:**
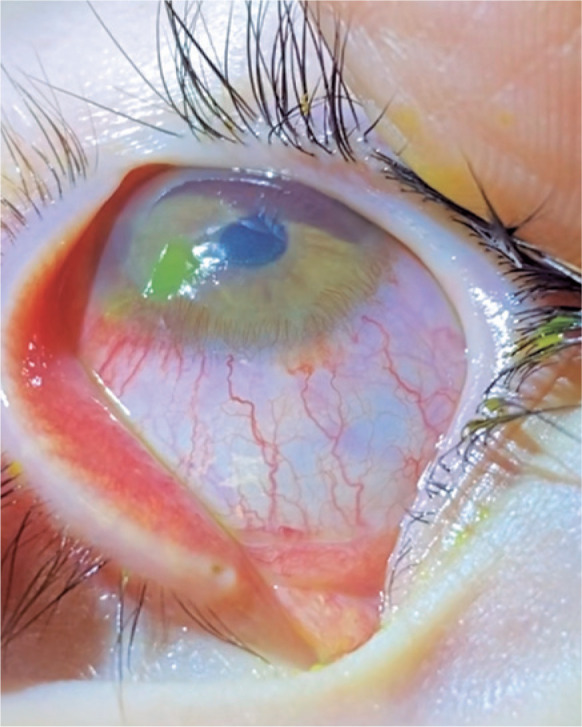
Same right eye after seven days of treatment, presenting partial epithelial defect with limbic insufficiency in the nasal and inferior regions of the cornea.

In the first case, ABHS was used by a 3-year-old girl before entering a park. The girl stepped on an ABHS dispenser pedal to sanitize her hands and the ABHS jet sprayed into her right eye, causing immediate ocular pain and burning. Her eyes were washed immediately with running water. Two hours later, the patient was admitted to an ophthalmologic emergency room, where copious irrigation was performed, and antibiotic eye drops were prescribed. The following day, an ophthalmological examination showed a visual acuity of 20/40 in the right eye and severe diffuse conjunctival hyperemia with an 80% cornea epithelial defect (Figure 1). Treatment was performed using a therapeutic contact lens, topical corticosteroids, antibiotics, lubrication, and oral vitamin C. After three days, her visual acuity was 20/200. The cornea showed Descemet folds and edema and was anesthetic. After 7 days, re-epithelialization was observed, but the patient sustained limbic insufficiency in the nasal and inferior regions (Figure 2). Three weeks later, the cornea presented complete re-epithelialization with superficial corneal neovascularization, and the patient’s visual acuity was 20/40. She currently maintains superficial neovascularization, corneal pannus, and residual astigmatism.

In the second case, a 3-year-old girl stepped on an ABHS dispenser pedal to sanitize her hands and the alcohol jet sprayed into her right eye, causing photophobia, ocular pain, and burning. She was immediately treated at an eye emergency unit, and antibiotics plus steroid eye drops were prescribed along with a therapeutic contact lens and systemic analgesics. A large nasal corneal ulcer and conjunctival ulcer were present. After 2 weeks, the cornea presented complete re-epithelialization, and the patient had normal vision of 20/20.

Both patients in this report experienced an inadvertent toxic concentration of an alcohol-containing product in the eye, leading to corneal and conjunctival ulcers. Patient 2 presented a typical clinical response after alcohol trauma, but the more chronic lesions in case 1 suggests the possibility of injury caused by other substances also present in the product, such as glycerol and hydrogen peroxide^(4)^.

The two girls each had easy access to ABHS dispensers that caused the accidents. These new dispensers store ABHS at a height of 100 cm with a pedal designed to facilitate hand hygiene in adults. However, these dispensers can cause ocular surface injury in children because the dispenser nozzle is at the height of their eyes.

Recently, Babić et al. analyzed Croatian Poison Control Centre data related to poisonings with disinfectants and hand sanitizers^(5)^. They identified that lesions due to exposure to hand sanitizers were more common in preschool children compared to adults^(5)^. These data conform to our report and show that dispensers of hand sanitizer should not be accessible by children.

Fortunately, the patients in this report have recovered their visual acuity and corneal re-epithelization after three weeks. Nevertheless, ABHS accidents in children require urgent attention. Health authorities should be aware that adequate control of these products is necessary, in addition to warning about the possible risks of their use.
